# First contact physiotherapists: are they able to reduce the burden on rheumatology services? A critical review of the evidence base

**DOI:** 10.1093/rap/rkad109

**Published:** 2023-12-09

**Authors:** Sarah R Golding, Jo Jackson

**Affiliations:** School of Sport Rehabilitation and Exercise Sciences, University of Essex, Colchester, UK; Rehabilitation Department, Southend University Hospital, Mid and South Essex NHS Foundation Trust, Westcliff-on-Sea, UK; School of Sport Rehabilitation and Exercise Sciences, University of Essex, Colchester, UK

**Keywords:** first contact physiotherapy, rheumatology, early referral, primary care

## Abstract

First contact practitioners have emerged over recent years in response to growing pressures within the National Health Service (NHS) and are now central to primary care musculoskeletal (MSK) services. Within the MSK field, these allied health professionals can be from a range of disciplines, including physiotherapy, podiatry and osteopathy. Early referral to rheumatology is key to successful long-term management of many inflammatory MSK conditions, but presents challenges to overburdened services. Evidence supporting the recognition and referral of patients with rheumatological disorders by First Contact Practitioners is lacking; however, physiotherapists have been shown successfully to substitute the role of a doctor within the MSK field. This review investigates the value of First Contact Physiotherapists (FCPs) within primary care and their role in early recognition and referral of rheumatological MSK disorders in line with national guidance. FCPs best placed to fulfil the role of MSK champions, positively impacting the whole MSK pathway, with the potential to reduce the burden on rheumatology services. Planned rapid upscaling of FCPs over the next few years will support sustainability of MSK NHS services.

Key messages

- Early recognition and referral of inflammatory musculoskeletal conditions to rheumatology is key for successful long-term management.
- Primary care clinicians need to ensure appropriate timely referral, adhering to national guidelines.
- First contact practitioners can reduce burden on rheumatology services through streamlining patient care through appropriate musculoskeletal pathways.

## Introduction

FCPs are physiotherapists with advanced clinical practice skills who are able to assess, diagnose, treat, and discharge without medical input, they are competent at managing the full spectrum of MSK patients.

  ([1], p. 3)

The advent of FCP practice in 2014 saw advanced level musculoskeletal (MSK) physiotherapists moving into primary care to support general practitioner (GP) shortages and a looming crisis within the National Health Service (NHS) [2]. In the UK, 90% of all clinical contacts take place in primary care, which is considered the bedrock of the NHS, MSK conditions make up ∼20% of the workload of GPs [3, 4]. The NHS long-term plan (2019) [5] promotes increasing diversity of specialities within primary care, with patient-centred care at the heart. Placing MSK specialist physiotherapists within primary care ensures appropriate expertise leading the patient pathway, with patients seeing the right person in the right place at the right time [6].

Demonstrable benefits of the FCP service include reduction in waiting times, improved quality and speed of treatment and recovery, increased self-management, reduced inappropriate referrals to secondary care, reduced GP workload and reduced pressures on NHS services [1]. Recent NHS funding for primary care networks through the additional role reimbursement scheme has seen a rapid increase in FCPs within primary care over the last 4 years [7], with the latest NHS workforce plan detailing extension of this [8].

The FCP role is an example of task shifting within health care, involving the matching of skills to changing needs of the NHS. Task shifting was traditionally conceptualized by the transfer of responsibility for simple tasks to less skilled workers with lower pay, for economic gain and improved efficiency [9]. It is now widely accepted that with certain tasks, e.g. MSK assessment and management, substituting an alternative clinician, in this case a physiotherapist instead of GP, is sensible. Innovation of such new roles involves careful planning and preparation, with adequate training and governance frameworks.

There are >200 rheumatological conditions, ranging from various types of arthritis to systemic CTDs and bone conditions, affecting one-third of people of all ages during their lifetime [10]. It is therefore not surprising that rheumatology services are overburdened. The British Society for Rheumatology (BSR) workforce report of 2021 highlighted that only a minority of rheumatology departments in the UK currently meet the staffing recommendations of one rheumatology consultant and a specialist nurse for every 60 000–80 000 population [11].

Management of inflammatory conditions has undergone a paradigm shift over the last 10–15 years, with emphasis now on early intensive medical management [12]. A 12 week window of opportunity from onset of symptoms to treatment has been evidenced to achieve remission, prevent joint and organ damage, reduce mortality and improve quality of life, with diagnostic delays having a negative long-term impact on patients [12–16]. However, early recognition is extremely challenging, given the rarity and heterogeneous nature of pathologies [17]. No clinical tests are 100% sensitive and specific, and a lack of positive signs on laboratory tests does not rule out inflammatory pathology [18]. Astoundingly, axial spondyloarthritis (axSpA) takes on average 8 years to diagnose [19] in the UK; the national campaign, ‘Act on Axial SpA’, launched in 2021 aims to reduce average time for diagnosis to 1 year [12, 20, 21]. FCP roles have been proposed to reduce these diagnostic delays [22].

The National Early Inflammatory Arthritis Audit (NEIAA) published its fourth annual report in 2022, reporting that 54% of patients were referred within the recommended time frame, and 39% of those referred received a diagnosis of early inflammatory arthritis [12]. This represents an increased conversion rate from previous years, suggested to reflect improved triage, referral pathways and awareness of symptoms. Key recommendations of the NEIAA include training for primary care staff (including FCPs) and exploration of triage mechanisms resulting in more appropriate and timely referrals [12]. FCPs and MSK physiotherapists were mentioned specifically in the NHS England Getting it Right First Time (GIRFT) initiative, tasked with promoting direct referrals from them [12, 23].

This review explores the value of FCPs within primary care in reducing the burden on rheumatology services through appropriate, high-quality and timely referral to the speciality.

## Methods

**Figure 1.**
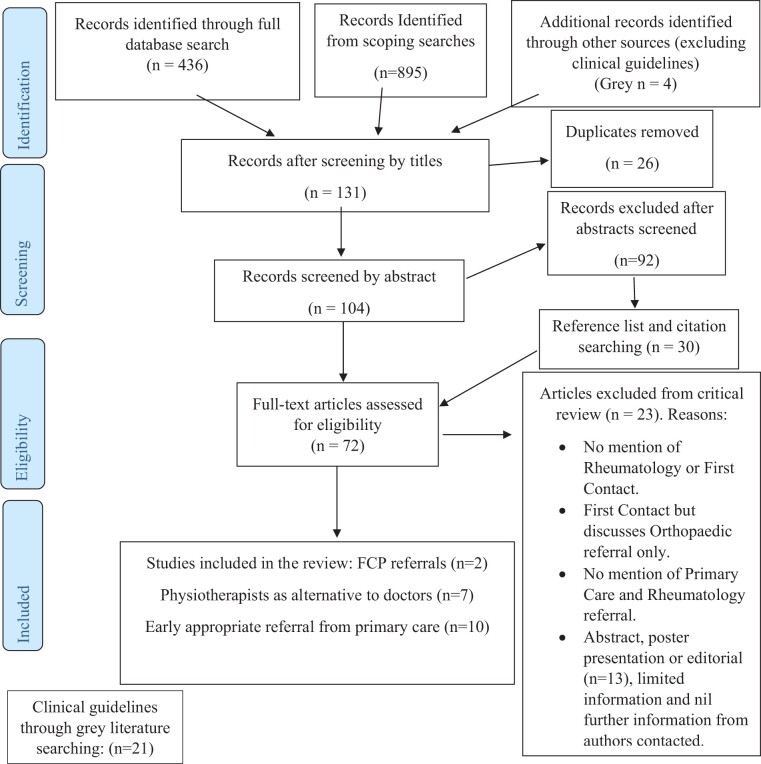
PRISMA flowchart detailing the search and selection process of evidence for the review

A comprehensive process was used to search the evidence base. Search terms were derived using the PICO framework: the population, patients with rheumatological conditions; the intervention, FCP; the comparison, primary care; and the outcome, secondary care referral. Relevant databases including AMED, CINAHL, EMBASE, EMCARE, Medline and PubMed were searched. Studies of any design published in the last 15 years (2008–2023) and written in English were included. Extensive grey literature searching was conducted, which involved contacting key professionals in the field and special interest groups. The search strategy is detailed in a modified PRISMA flow diagram (Fig. 1).

Only one published study evaluating FCP referrals to rheumatology services was identified [24], and one other study evaluating a model FCP service [25]; the relatively recent emergence of the FCP role is the likely reason for this. Search parameters were therefore widened to include evidence relating to key themes: physiotherapists as an alternative to doctors in assessing MSK disorders [26–32]; rheumatology clinical guidelines (establishing thresholds for referral) [18, 33–52]; and early appropriate referral of rheumatological conditions from primary care [17, 22, 53–60]. The aim, to establish and propose the value of the role in reducing the burden on rheumatology. Studies were analysed critically using the critical appraisal skills programme tools [61].

## FCP referrals

There is limited evidence supporting physiotherapists as primary assessors (first contact) of rheumatological conditions and safety of the FCP role; only two UK (Scotland) published studies exist [24, 25]. Hepburn [24] presents audit data over a 3 year period (2019–2022) evaluating referrals of axSpA from advanced practice physiotherapists working as the first contact in primary care. The author reported a significantly lower mean time (3.4 years) to diagnosis than the reported UK average (8.5 years) [12, 20, 21] and that 78.9% of referrals were compliant with National Institute for Health and Care Excellence (NICE) guidelines and the SPADE tool criteria [24]. Two-thirds of patients receiving a positive diagnosis of axSpA fulfilled the referral criteria, demonstrating the heterogeneous nature of presentation. Diagnostic conversion rates were noted to be comparable to previous studies of medical staff in general practice [24, 62].

Physiotherapists within both studies had a suitable level of experience (Band 7 and 8a Agenda for Change) as set out by the NHS and Chartered Society of Physiotherapy (CSP) implementation guidance for FCP roles [63–65]. Downie *et al.* [25] presented a 2 year service evaluation, convincingly establishing benefits of physiotherapists as an alternative to GPs in assessing and managing MSK conditions within primary care. Reported referral rates onto ‘other secondary care services’, including rheumatology (and other specialities), were 0.6%; other case studies concur with this (˂1%), although no specific rheumatology referral statistics exist in the literature [25, 64, 66, 67]. The authors commented that their service evaluation did not include a safety audit, but they were not aware of any missed diagnoses. Hepburn’s study [24] did not evaluate missed cases of axSpA but reported a higher incidence of cases compared with that previously cited in the general population. Very few studies evaluate re-referral or re-presentation rates, but of those published none identified missed serious pathology or inflammatory pathologies [32, 68, 69].

## Physiotherapists as an alternative to doctors in assessing MSK disorders

A strong evidence base exists to support the concept that physiotherapists can provide equal or superior care to a doctor when assessing and managing MSK conditions [24, 28–32, 69–76]. Ludvigsson and Enthoven’s Swedish-based study supports physiotherapists as primary assessors of MSK conditions [31]. The authors evaluated safety, reporting that potential serious pathologies identified by physiotherapists were confirmed by GPs; furthermore, patients who decided to return to their GP for the same problem for which they had seen the physiotherapist had no serious pathologies. It was therefore concluded that physiotherapy primary assessment was safe [31]. This study was conducted between 2004 and 2007, a decade before the advent of the FCP role in the UK; indeed, the role is still not established across Europe.

Physiotherapist-led MSK triage or clinical assessment and triage services are commonplace within health care, traditionally situated within secondary care or as an interface between primary and secondary care. Their purpose is to triage and rapidly assess patients with MSK conditions referred to secondary care, facilitating access to treatment, improving efficiency and reducing inappropriate referrals [72, 75]. Rheumatology triage has been researched thoroughly in Canada, where training and development of unique ‘Advanced Clinical Practitioners in Arthritis Care’ (ACPAC) was driven by a shortage of rheumatology consultants, and much can be learnt from this model [26, 27, 29, 30, 70, 77]. Triage is performed by an experienced clinician directing patients in a timely appropriate manner either to a rheumatologist for management of inflammatory arthritis or to physiotherapy and other services for non-inflammatory conditions. Patients see the right person, in the right place; FCP builds on this by creating one less step in the pathway, enabling patient to see the right person first time, thereby reducing duplication and pressures on secondary care [64].

Triage studies have shown high correlation between consultant and physiotherapist diagnosis within orthopaedics [28, 32, 73, 75, 76] and rheumatology [24, 26–30, 32, 69, 71]. Concordance of diagnosis of inflammatory arthritis between physiotherapists and rheumatologists is documented to range from 89% [71] to 91% [26]. Studies with lower accuracy rates involved less experienced physiotherapists [27], not representative of the level of experience of FCPs within the NHS, who are Band 7-8a ‘Agenda for Change’ (AFC) [63–65].

Studies examining the efficacy of physiotherapists within advancing roles (substituting doctors) must be interpreted with caution where competency is unknown [28, 31, 76]. Professional scope of practice varies between countries and often is not described in detail within the literature; attributing success of the role to the level of expertise of the clinician is therefore impossible [76]. A recent UK survey reported the majority of FCPs had extended skills, including ordering of investigations [78]; this is consistent with latest CSP FCP service principles (2021), which state that FCPs should have, as a minimum, the same referral rights as GPs, including diagnostics [79].

Clearer standards and career pathways in the UK are now emerging to ensure safety, success, development and longevity of the FCP role, including: ‘Roadmap to Practice’ [6] and ‘Principles of effective and sustainable FCP service’ [79]. Documents explicitly state that clinicians are examined against competencies at level 7/master’s level [66, 79], including advanced knowledge of assessment, diagnosis and management of inflammatory (non-mechanical) disorders. MSc pathways in Higher Education Institutes are responsive to this.

## Rheumatology clinical guidelines

Many strategies have been developed to assist early appropriate referral, including national evidence-based guidelines published by NICE [18, 36–40] and BSR [33, 46–48, 50, 51], electronic tools, standardized referral forms and various campaigns [18, 20, 23, 33, 36, 80]. The NEIAA measures metrics of care against the NICE Quality Standard 33 [36] for RA; additional specialist guidelines and resources exist for other common rheumatological conditions, including those developed in 2022 by the Best MSK Health Collaborative and GIRFT [34, 35, 40, 43, 52]. More recent guidance goes one step further, suggesting, with the exception of emergency pathways (e.g. GCA), that all referrals to rheumatology should be made via specialist ‘Advice and Guidance’ routes [81].

Key guidelines and standards within rheumatology are available through NICE [18, 36–40], EULAR [44, 45, 49] and BSR [46–48, 50, 51]. NICE guidelines provide the UK standard, BSR guidelines are commonly accredited by NICE, and EULAR guidelines provide a European standard, but so many guidelines can muddy the waters. In response to this, the BSR developed the ‘Adult Rheumatology Referral Guidance’ [33], which combines guidelines, simplifying and clarifying referral thresholds, and outlining key signs and symptoms of common rheumatological conditions.

The NEIAA evaluates national practice against NICE Quality standard (QS33); RA in over 16s [36], which explicitly states that referral should be initiated within ‘3 working days of presentation of symptoms’. The 2022 audit revealed that 54% of patients with inflammatory arthritis were referred within the appropriate time scale [12]. Fifty-one per cent of patients referred were confirmed to have inflammatory arthritis; this included 32% RA, 8% PsA, 9% undifferentiated arthritis and 2% axSpA. This 2022 statistic does not include conditions referred such as CTDs or vasculitides and other rare systemic disorders with MSK manifestations; however, the principles of early diagnosis and correct pathways are alike. The 2023–2024 NEIAA is collecting data for these conditions.

Deciding on whether to refer or not requires sound clinical reasoning and awareness of guidelines, balancing sensitivity, specificity and positive likelihood, in order to detect the majority of patients with rheumatological conditions without overburdening rheumatology services [53]. Recognition and referral of axSpA continues to challenge clinicians, with delays to diagnosis being much greater than with inflammatory arthritis [12, 82]. NICE recognizes this, providing referral criteria for SpA that are similar in construction to the SPADE tool (originally validated for use in secondary care) [83]. Despite their use to identify potential cases, sensitivity and specificity in a recent study were found to be 66.7 and 21.4, respectively, highlighting heterogeneity of presentation [24]. The recent national campaign ‘Act on Axial SpA’ presents a target of 1 year maximum from symptom onset to diagnosis [20], and an 8 week target from presentation to assessment within an axSpA specialist clinic is advocated [34].

Clinical care pathways enable local application of guidelines depending on the regional population and resources, with the aim of ensuring high-quality patient care [84]. These pathways need to be transparent to all stakeholders and involve engagement and collaboration between primary and secondary care [15, 23]. For example, guidelines state that GCA, a medical emergency, should be treated immediately with CSs and referred to a specialist for evaluation on the same working day (ideally) or within 3 working days [50]. In reality, this means referral to Accident & Emergency or via fast-track pathways into rheumatology; however, one-third of NHS trusts nationally have no formal pathway, resulting in more than half of patients not meeting the guideline [23]. FCP implementation guidelines advise integration of FCP services within the surrounding MSK system and clear lines of communication, thereby linking primary to secondary care [79]. FCPs working across sectors are able to promote local pathways (and national guidelines), ensuring that primary care clinicians have the knowledge to implement best practice.

## Early, appropriate, quality referral from primary care

The concept and definition of appropriate referral is challenging to define, Downie *et al.* describe appropriateness as ‘investigations and/or treatment only available in secondary care setting, or a review or open appointment being given’ ([25] p. e316).

A recent American study concurred with this: ‘if no continuing care was offered, then referral was not appropriate’ ([85], p.3). This definition, although broad, is consistent with guidelines and reflects that not every rheumatological condition requires referral to specialist services; some can be effectively managed in primary care (e.g. gout, PMR, osteoporosis). Inflammatory arthritis and the rarer autoimmune diseases (CTDs and vasculitides) are often complex to diagnose, representing the core work of rheumatology, and therefore should always be referred [23, 81]. Conversion rates to orthopaedic intervention (i.e. surgery) dominate FCP literature [25, 64, 67]. Hepburn’s study [24], however, shows a high conversion rate to further investigations (spinal MRI and HLA-B27 testing), supporting concordance of impressions between the physiotherapist and rheumatologist.

Accurate early referral of patients with inflammatory arthritis from primary care is the ultimate goal, enabling formal diagnosis and initiation of treatment within the 12 week window of opportunity, with the aim of disease remission [12]. A recent UK study found that this is achieved in only 20% of patients with RA and that many visit the GP (and, less frequently, other health-care professionals) multiple times before rheumatology referral is initiated [17, 58, 60]. Effective FCP services should achieve this by placing expertise at the beginning of the patient pathway. Delays in referral and initiating early management of inflammatory arthritis occur at various stages of the patient journey; primary care is a crucial stage representing the longest delays, with GPs traditionally acting as the gatekeepers for secondary care [17, 19, 55, 57, 59]. Key studies provide useful insight into the challenges of early referral from primary care [54, 56–59].

The national survey by Scott *et al.* [56] found that the majority of GPs requested investigations before considering referral. NICE standards state that investigations can be initiated at the time of referral but should not influence the decision of whether to refer or not [4, 36, 37]. Tests can both falsely reassure clinicians or falsely raise suspicion of pathology [56]; this is true for RF, which as an isolated test has low specificity and sensitivity for inflammatory arthritis and without the presence of positive inflammatory markers or symptoms is not diagnostic [4, 52, 56]. Likewise, elevated inflammatory markers (ESR and CRP) and positive HLA-B27 do not rule axSpA in or out despite emphasis on these by clinicians [18, 39, 53, 54]. One case review found significant delays for patients with inflammatory arthritis who had undergone radiographic investigations; conventional radiography is known for its low sensitivity in detecting joint damage [44]. However, results need to be interpreted with caution because of the small sample size and large proportion of missing data owing to incomplete medical records [54].

Referral of inflammatory arthritis should be based on presenting signs and symptoms irrespective of investigations; however, the heterogeneous nature of the pathology, relative rarity amongst MSK conditions and challenges of identifying persistent synovitis impede this [4, 17, 37, 59]. General practitioners within qualitative studies liken identification of inflammatory arthritis to finding a needle in a haystack and express their uncertainty regarding whether to trust laboratory tests or clinical features more [59]. Causation has been viewed as multifactorial, thereby proving difficult to address (disease characteristics, patient characteristics, lack of definitive tests, system factors, clinician knowledge and experience) [58, 59, 86].

NICE (and EULAR) guidance defines key features of synovitis, adding detail to guidelines using expert opinion and evidence [36, 45]; key studies reviewed show a significant association between clinician experience and confidence in diagnosing inflammatory arthritis, which is not surprising [54, 56, 59]. The large UK survey by Scott *et al.* [56] had a good response rate, enabling generalization of results, and their findings add meaning and depth to NEIAA [12] by exploring the opinions and views of clinicians. Clinicians need to place less weight on investigations and more on early referral based on presenting symptoms, but this requires a paradigm shift and represents a careful balancing act relying on knowledge and experience of the clinician [4, 56]. The decision by physiotherapists to refer patients within one study investigating axSpA was made on clinical assessment and X-ray imaging, without blood tests and MRI owing to lack of access [24]. Results support advanced clinical reasoning skills of physiotherapists without over-reliance on investigations which, in most confirmed cases of axSpA, were in fact negative.

Chronic back pain is very common across populations, constituting 3–7 million GP consultations in the UK annually. Primary care is the most common first point of contact for these patients, and an estimated 5% have axSpA [82]. Misdiagnosis of mechanical back pain is common owing to similar behaviour of symptoms; furthermore, axSpA is low in the list of differentials and perceived as uncommon [22, 53, 86]. The survey by Gregory *et al.* [55] into diagnostic delay found that a large proportion (63%) of patients with axSpA visited their GP on one or more occasions before being referred to secondary care, and 14% of patients underwent >10 visits. Physiotherapists were less frequently visited, but also contributed to delays. In contrast, a recently published national audit reported a marginally higher level of visits to physiotherapists than to GPs; however, experience levels were not evaluated [19]. To drive change, the National Axial Spondyloarthritis Society (NASS) identified that axSpA needs to be higher in the clinical reasoning of primary care clinicians, including MSK physiotherapists, who have been shown to lack awareness, knowledge and confidence in screening cases [22, 53, 55]. Key features of axSpA are elucidated only through questioning [18, 39, 83], although it has been suggested that this routine questioning is not core practice in back pain assessments [53]. This requires time, which a busy primary care environment does not lend itself to, and respondents (GPs) in one study reported an average of 15 min for a consultation [86].

Proficient clinical reasoning within MSK practice is a complex process developed through years of experience. Physiotherapists, as MSK specialists, should be skilled in recognition of axSpA. Surveys by Steen *et al.* [22, 53], however, showed a lack of awareness of screening and referral of suspected cases, which is worrying, especially considering that safety is crucial when working within first contact roles. Physiotherapists with greater experience (i.e. higher banding) and those in FCP roles demonstrated higher diagnostic accuracy, as one would expect [22]. FCPs also had greater knowledge and awareness of referral guidelines, which might reflect targeted education over recent years with the introduction of the ‘Roadmap to Practice’ [6]. Despite this, continued education and training for FCPs and MSK physiotherapists is needed [22]. The Rheumatology Physiotherapy Specialist Interest Group have responded to this by publishing the ‘National Rheumatology Physiotherapy Capabilities Framework’ (endorsed by the CSP and BSR), the first of its kind [87].

With the global challenge of timely access to rheumatology services owing to increasing pressures and an insufficient workforce [11], it is paramount that non-inflammatory MSK conditions (predominantly OA and FM) are identified early in the pathway and directed to community MSK or interface services, which are better suited to fulfil patient needs [11, 12, 15, 23, 88]. Referral of these to rheumatology only increases wait times for inflammatory disorders [81].

Delays in secondary care in addition to primary care have been reported, with some patients undergoing multiple rheumatology appointments before establishing a formal diagnosis [17, 54, 55]. These delays have been attributed to atypical presentations, with involvement of fewer joints, proximal joints and negative RF and anti-CCP [89, 90]. High numbers of referrals have also been shown to be attributable to GP uncertainty and concerns over missed diagnosis [23, 59]; despite this, primary care clinicians filter out huge numbers of patients quickly and efficiently, many with multiple non-inflammatory joint pains.

Atypical presentations exhibiting some characteristic signs and symptoms of inflammatory conditions but not fulfilling all referral criteria present uncertainty for clinicians. These so-called ‘grey area’ cases might warrant referral for expert opinion but also increase waiting lists, lack of access to specialist advice in these and other cases has been proposed to delay diagnosis further [17]. Triage services are shown to be useful in addressing these cases. Forgie *et al.* [91] present service data for an initiative in the UK whereby Forgie, a GP with rheumatology special interest, triaged grey area referrals, adding additional information from primary care notes, ordering additional investigations and conducting face-to-face assessments. Further information was deemed necessary in more than two-thirds of referrals; 40% of patients deemed from the information to have non-inflammatory conditions were confirmed in most cases through physical assessment, and the majority of these received a diagnosis of FM and were directed to appropriate services. Clinical specialist physiotherapists have been shown to fulfil such roles, diverting large numbers of non-inflammatory conditions away from rheumatologists, who are able to dedicate their time to inflammatory and complex conditions [68, 69, 71, 88, 92]. This is a role that has been evidenced to work within and outside the UK [26, 27, 29, 69–71], development of which is recognized as one answer to the workforce crisis and supported by the recent introduction of the Rheumatology Physiotherapy Capabilities Framework [87].

Adequate referral information is required to allocate patients effectively to specific clinics and pathways in secondary care; furthermore, the right work-up (investigations) saves valuable time, enabling diagnosis and appropriate management to be initiated early. Wong *et al.* [93] evaluated referral quality of >2000 letters from primary care by checking information including; the reason for referral, medical and family history, diagnostic tests and symptoms. Incomplete pertinent information was found, even with a lack of basic details such as symptom description and duration. Inevitably, patients can be triaged to the wrong service, experience increased waiting times to diagnosis, wasted time and resources, and a delay to appropriately referred patients [23, 93]. This Canadian study has a moderate cross-over relevance to UK practice; therefore, it is valid in the context of this review alongside discussion of UK practice. Limited time within busy primary care does not lend itself to formulation of detailed referrals; FCPs have on average 20 min per consultation, which is more than that of GPs [78]. Standardized referral forms are one solution recommended by GIRFT [23] and EULAR, with the need for clear referral criteria and request for sufficient pertinent details [45]. Currently, a national form does not exist, and there are huge variations in referral management systems [23, 93].

Solutions to aid timely and appropriate referral from primary care to rheumatology have been proposed, including education programmes, screening tools, improved referral guidelines and development of clinical decision aids [4, 12, 17, 23, 55, 57–59, 80], and current examples of these within UK clinical practice exist [15]. ‘Advice and Guidance’ has been encouraged as a valuable source of specialist opinion with potential to relieve pressures on rheumatology services; there is, however, recognition of its slow uptake and the need for allocated rheumatologist time [12, 23]. Of note, from March 2021 the electronic referral service, via which ‘Advice and Guidance’ requests are made, allowed specialists to convert queries straight to a referral if clinically indicated [81]. The BestMSK Health Collaborative ‘High Impact Restoration Strategy’ has proposed that all referrals to rheumatology (except emergencies) should come via this advice route [94]. Successful implementation of routine use of ‘Advice and Guidance’ might be a game changer, the BSR have recently developed resources to assist this [95].

## Conclusion

This review gives insight into FCP practice and their referral of patients to rheumatology services. Evidence to support FCPs as effective primary assessors of rheumatological conditions is lacking; however, it is clear that with the right level of skill and experience this role has the potential to impact positively both the patient and rheumatology services. More emphasis is required to yield evidence, not only to support the role but to advance practice. As upscaling of FCP services continues over the next few years [65, 96], a larger proportion of the primary care MSK caseload will be managed by FCPs, resulting in greater impact on MSK pathways, optimum patient care and judicious use of limited NHS resources. FCPs are ideally placed as MSK champions, bridging the gap between primary and secondary care. With the rapid upscaling of FCPs in primary care proposed over coming years, FCPs could have real impact.

## Data Availability

No new data were generated or analysed in support of this review.
